# Annual Announcement of the Medical Department of Penn. College

**Published:** 1851-10

**Authors:** 


*Annual Announcement of the Medical Department of Pennsylvania College. Session* 1851-2.

WE learn from the above announcement, that the lectures in the medical department of this institution, will commence on Monday, 13th of

October, and continue until the first of March. Besides the regular lectures, clinical instruction will be given in the Pennsylvania hospital, by the physicians and surgeons of that institution, and in the college, by the Professors of the Principles and Practice of Medicine and the Principles and Practice of Surgery.

